# Impact of Indocyanine Green Fluorescence Imaging on Lymphadenectomy Quality During Laparoscopic Distal Gastrectomy for Gastric Cancer (Greeneye): An Adaptative, Phase 2, Clinical Trial

**DOI:** 10.1245/s10434-023-13848-y

**Published:** 2023-07-13

**Authors:** Carlo Sposito, Marianna Maspero, Valeria Conalbi, Andrea Magarotto, Michele Altomare, Carlo Battiston, Paolo Cantù, Vincenzo Mazzaferro

**Affiliations:** 1 Upper GI Surgery and HPB Surgery and Liver Transplantation Unit, Fondazione IRCCS Istituto Nazionale Tumori, Milan, Italy; 2 Department of Oncology and Hemato-Oncology, University of Milan, Milan, Italy; 3 Gastroenterology and Digestive Endoscopy, Fondazione IRCCS Istituto Nazionale Tumori, Milan, Italy; 4 Department of Surgical Sciences, Sapienza University of Rome, Rome, Italy

## Abstract

**Background:**

Indocyanine green (ICG)-guided lymphadenectomy using near-infrared visualization (NIR) may increase nodal yield during gastrectomy. The purpose of this study was to evaluate the clinical benefit of NIR visualization on the quality of D2 lymphadenectomy during laparoscopic distal gastrectomy.

**Methods:**

This single-arm, open-label, Simon’s two-stage, adaptive, phase 2 trial included patients who underwent laparoscopic distal gastrectomy for gastric adenocarcinoma. Endoscopic peritumoral injection of ICG was performed 24 ± 6 h before surgery. Intraoperatively, after standard D2 lymphadenectomy and specimen extraction, NIR was used for eventual completion lymphadenectomy. The primary endpoint was clinical benefit of NIR (i.e., at least one additional harvested station containing lymph nodes, with negative points for every harvested station with no lymph nodes at final pathology).

**Results:**

We enrolled 18 patients (61% female, median age 69 years). With NIR, an extra 23 stations were harvested: 9 contained no lymph nodes, 12 contained nonmetastatic lymph nodes, and 2 contained metastatic lymph nodes. The most commonly visualized station with NIR were station 6 (8 patients) and 1 (4 patients). The total number of harvested nodes per patient was 32 (interquartile range [IQR] 26–41), with a median of 1 (IQR 0–1) additional lymph node after NIR. Overall, seven (39%) patients had a clinical benefit from NIR, of which two (11%) had one metastatic lymph node harvested with NIR.

**Conclusions:**

NIR visualization improves the quality of D2 lymphadenectomy in distal gastrectomy for gastric cancer. Considering the limited improve in the number of harvested lymph nodes, its real oncological benefit is still questionable.

**Supplementary Information:**

The online version contains supplementary material available at 10.1245/s10434-023-13848-y.

Despite advances in systemic therapies and neoadjuvant strategies, the mainstay curative intent treatment for resectable gastric adenocarcinoma remains gastrectomy with lymphadenectomy.^1^ The extent of lymphadenectomy recommended by European, American, and Japanese guidelines is a D2 lymphadenectomy, because it provides more accurate tumor staging and improves survival outcomes.^2–7^ Currently, lymphadenectomy is performed according to anatomical landmarks with no nodal visualization method, and its accuracy is largely dependent on surgical expertise. Performing an adequate lymphadenectomy without increasing the risk of perioperative complications remains a challenge.

Indocyanine green (ICG) is a fluorescent dye that can be visualized in the near-infrared (NIR) spectrum. Thanks to its excellent safety profile and fluorescent properties, ICG has several uses in the medical field. ICG has been successfully utilized for sentinel lymph node (SLN) visualization in different areas of surgical oncology, including urology, gynecology, and breast surgery.^8^ In addition to SLN, injection of ICG in peritumoral tissues also may allow the intraoperative visualization of lymphatic patterns and could be used as a guide for lymphadenectomy.

The utility of NIR-visualization for D2 lymphadenectomy in gastric cancer is currently under investigation. A recent meta-analysis has shown a mean increase of 7 (95% confidence interval [CI] 1.37–12.67) lymph nodes retrieved during NIR-guided D2 lymphadenectomy compared with standard visualization.^9^ Similarly, ICG-guided D2 lymphadenectomy has been associated to reduced lymph node noncompliance (i.e., absence of lymph nodes from a retrieved nodal station at final pathology).^10,11^ Despite promising results, ICG-guided D2 lymphadenectomy is still far from becoming a standard of care. Most of the evidence in this field is based on retrospective and cohort studies, with only two published, randomized, open-label, controlled trials (RCTs).^10,12^ These latter studies reported a significant increase in the number of lymph nodes when performing NIR guided D2 lymphadenectomy: despite level 1 evidence, the magnitude of the effect might be partially influenced by a “performance bias,”^13^ typical of open label RCTs on interventional procedures.

In this study, we conducted a single-arm, Simon’s two-stage, phase II trial designed to prospectively assess the clinical benefit of NIR visualization in D2 lymphadenectomy during laparoscopic distal gastrectomy for gastric adenocarcinoma. To avoid performance bias, we planned to use NIR visualization only at the end of standard “blind” D2 lymphadenectomy to evaluate the pure impact of the technique. We considered retrieval of nonnodal tissue (nodal noncompliance) as a potentially harmful event, and in each patient, NIR was considered useful only if the number of additional retrieved nodes was higher than the number of nonnodal tissues.

## Methods

### Study Design

This was a single-center, phase 2 trial using a Simon’s optimal two-stage design^14^ to evaluate the impact of NIR visualization after ICG injection on the quality of D2 lymphadenectomy during laparoscopic distal gastrectomy for gastric adenocarcinoma. The primary endpoint was the clinical benefit of NIR, defined as at least one additional harvested station containing lymph nodes, with negative points for every harvested station with no lymph nodes at final pathology, as described in the following paragraphs.

The study was approved by the institutional review board. It was conducted in accordance with the Declaration of Helsinki and according to Good Clinical Practice. All patients provided written, informed consent before enrolment. Subjects were enrolled between December 2020 and July 2022.

### Inclusion and Exclusion Criteria

Adult patients with histologically diagnosed resectable gastric adenocarcinoma who were candidate to curative-intent laparoscopic distal gastrectomy were eligible for the study. Inclusion criteria were (1) cT1b-T4, cN0-N+, M0 preoperative staging; (2) performance status 0–1 per the Eastern Cooperative Oncology Group (ECOG) criteria; (3) age 18 to 80 years. Exclusion criteria were (1) previous gastric resection, or endoscopic submucosal resection of early gastric cancer; (2) presence of lymph nodes > 3 cm at preoperative staging; (3) indication to total or proximal gastrectomy, including gastroesophageal junction cancers; (4) previous adverse reaction to ICG injection; (5) pregnant or lactating patients; (6) patients with absolute contraindications to surgery.

### Interventions

**Fig. 1:**
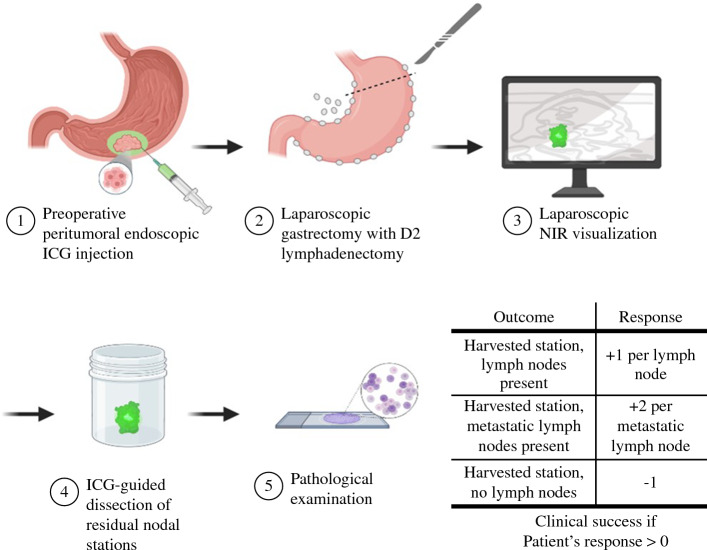
Study design

The study design is shown in Fig. 1. ICG was endoscopically injected around the tumor 24 ± 6 hours before surgery. ICG (Verdye^®^) was diluted in sterile water to 1.25 mg/ml, and a total of 2 ml were injected submucosally at the four cardinal points around the gastric lesion (0.5 ml per cardinal point).

Patients underwent laparoscopic distal gastrectomy with D2 lymphadenectomy according to international guidelines. Surgery was performed by two senior surgeons (CS or VM) with extensive experience (>30 interventions each) in laparoscopic surgery for gastric cancer.

Standard D2 lymphadenectomy was performed according to Japanese Gastric Cancer Association (JGCA) guidelines,^4^ with lymphadenectomy of stations 1, 3 to 7, 8a, 9, 11p, and 12a along the anatomical borders, without the use of NIR visualization. Lymphadenectomy and omentectomy are always performed en bloc with distal gastrectomy: in brief, after coloepiploic detachment, the gastrocolic ligament is divided and the greater curvature is skeletonized up to the gastrosplenic ligament. Right gastroepiploic vessels are ligated and dissected at the origin; the dissection continues following the gastroduodenal artery up to the right gastric vessels, which are ligated and dissected at the origin. The duodenum is then divided at least 1 cm distal to the pyloric sphincter with an endostapler. The lesser omentum is sectioned, lymphadenectomy proceeds with stations 12a, 8a, and 11p until isolation and dissection of the left gastric vessels at the origin, and then proceeds to station 9 up to the esophageal pillar. Station 1 node are lowered 5–6 cm below the esophagogastric junction. The proximal side of the stomach is divided at least 4 cm cranially from the tumor as previously described^15^ and the specimen extracted through a small Pfannestiel incision. Finally, latero-lateral gastrojejunostomy on the greater curvature is performed with Roux-en-Y reconstruction. Only after specimen extraction, NIR visualization was performed using the NOVADAQ SPY Elite system (Stryker^®^). For patients with primary tumor in the distal antrum, a partial Kocher’s manoeuver was performed to evaluate station 13. In case of visualization of fluorescent tissues either inside or outside the D2 field, those were dissected and sent to pathology. In case of fluorescence of extra D2 stations, the dissection involved only the visible fluorescent tissue and not the entire anatomical basin. Dissection of fluorescent tissue in extra D2 stations, while not routinely performed at our center in accordance with current clinical guidelines, was performed in the context of this study to check whether fluorescence corresponded with nodal tissue and, particularly, with metastatic nodal tissue.

The surgical specimen was prepared by the surgical team together with the pathologist. Each nodal station was individually separated according to JGCA definitions, as shown in Supplementary Fig. 1, and evaluated for the presence or absence of lymph nodes.

After surgery, follow-up was performed with physical examination, blood tests, and cross-sectional imaging with computed tomography every 3–4 months for the first 2 years and every 6 months for the subsequent 3 years, with variations in the timing of the first follow-up depending on receipt of adjuvant chemotherapy versus observation. Timing of endoscopic surveillance was decided on a case-by-case basis depending on stage.

### Statistical Analysis

A Simon's two-stage design was used.^14^ The following assumptions were made:

- ICG use would be justified if, after NIR visualization, an additional lymph node was removed; its use would be further justified if the additional lymph node was metastatic;
- ICG use would not be justified if no additional lymph-nodes after NIR visualization were removed;
- ICG use would have a potentially negative effect if after NIR visualization a tissue with no lymph nodes was harvested.

According to these assumptions, the utility/futility of the procedure was quantified with the values shown in Table 1. The response was considered positive if the sum of values per patient was > 0. Overall, we considered an absence of clinical benefit of preoperative ICG injection if less than 20% of patients would have shown a response > 0. Thus, the Simon optimal two-stage design tested the null hypothesis of less than 20% positive responses against the alternative hypothesis of more than 50% positive responses. Accordingly, a total of 18 patients (8 and 10 patients for stages 1 and 2, respectively) were required to evaluate the primary endpoint at 5%, one-sided, type I error, and 80% power:

- In stage I, if less than two patients had a positive response, the intervention would be considered futile, and the study would be stopped.
- In stage II, an additional ten patients would be accrued. If at least seven patients had a positive response, the null hypothesis would be rejected, and the intervention would be considered effective.

**Table 1:** Values to evaluate the response according to final pathology

After NIR visualization	Values
Harvested LN, metastatic	+ 2 per metastatic lymph node
Harvested LN, negative	+ 1 per lymph node
No differences in management	0
Harvested LN, no LN at pathology	− 1 per harvested station

Categorical variables were reported as number of cases and percentages. Continuous variables were reported as median and interquartile range. Chi-square test was used to evaluate differences in the rate of patients with NIR finding of nodal tissue and with NIR finding of nonnodal tissue according to different baseline characteristics. Overall survival (OS), cancer-related survival and recurrence-free survival (RFS) were calculated with the Kaplan–Meier method. Statistical analyses were performed with the IBM SPSS Advanced Statistics 24.0 package.

## Results

### Preoperative Characteristics

Between December 2020 and July 2022, 18 patients were enrolled in the study. The median age at surgery was 69 years (interquartile range [IQR] 58–71.5), and 11 (61%) were females. Half of the cohort had clinically nodal positive disease, and 11 (61%) patients underwent neoadjuvant therapy. Neoadjuvant therapy regimens were FLOT in five cases, mFOLFOXIRI in four cases, and tremelimumab and durvalumab in two cases. Preoperative characteristics are summarized in Table 2.

**Table 2:** Preoperative characteristics (stages I–II)

	*N* = 18
Age (year)	69 (58–71.5)
Female gender	11 (61.1)
BMI	23 (21–26)
ASA	
I	1 (5.6)
II	15 (83.3)
III	2 (11.1)
cT	
T1	1 (5.6)
T2	4 (22.2)
T3	11 (61.1)
T4	2 (11.1)
cN	
N0	9 (50.0)
N+	9 (50.0)
Preoperative location	
Body	9 (50.0)
Antrum	9 (50.0)
Preoperative histology	
Intestinal adenocarcinoma	3 (16.7)
Diffuse adenocarcinoma	11 (61.1)
Mixed adenocarcinoma	2 (11.1)
Missing	2 (11.1)
Neoadjuvant chemotherapy	11 (61.1)

Data are number (percentage) and median (interquartile range)

### Perioperative Data

All patients underwent endoscopic ICG injection the day before surgery. The method of injection was according to protocol in all cases. No complications associated with the procedure were recorded. There were no new findings at preoperative EGDS compared to previous preoperative staging.

Operative characteristics and postoperative data are reported in Supplementary Table 1. All patients underwent laparoscopic distal gastrectomy with D2 lymphadenectomy. No patient required conversion to laparotomy. The median time interval between ICG injection and intraoperative NIR visualization was 22.5 (22–23) hours, adequate in all cases.

### Primary Outcome Measure

In the first eight cases, response was > 0 in 4; thus, the study proceeded enrolling the subsequent ten cases. Overall, response was > 0 in 7 cases (38.9%), and the null hypothesis was rejected.

NIR visualization resulted in the following:

- ICG fluorescence was visible in 13 (72.2%) patients: in 12 (67%) patients in the D2 area and in six (33.3%) in the area outside D2 (station 13 in 2 patients, stations 2, 4sa, 11d, and 10 in one patient each).
- 23 extra stations were harvested (17 in the D2 area, 6 in the extra-D2 area), with a median of 1 (0–1) extra station harvested per patient.
- The most commonly visualized station with NIR was station 6 (8 patients), followed by station 1 (4 patients)
- 15 nodes were retrieved overall, less than one per patient, 2.3% of the total 641 nodes harvested. NIR did not change appropriateness of lymphadenectomy (in terms of number of nodes harvested) in neither patient
- Two metastatic nodes were retrieved overall, 2.5% of the total 78 metastatic nodes. One metastatic node was retrieved in a patient with 27 metastatic nodes, one in a patient with four metastatic nodes. NIR did not change the staging in neither patient.
- Retrieval of nonnodal tissue in nine instances in six patients. Additional retrieval of tissue did not result in any immediate complication.

**Fig. 2:**
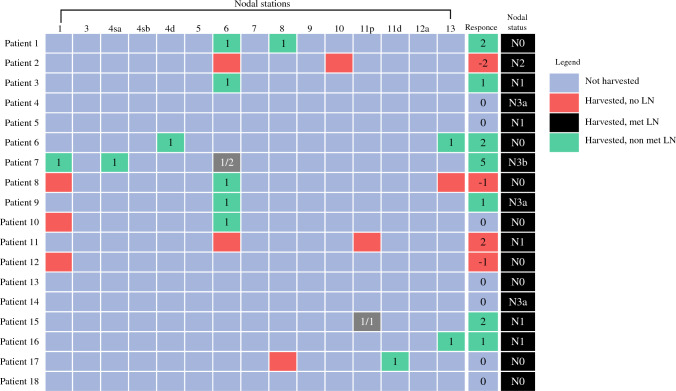
Harvested nodal stations after NIR visualization and presence or absence of lymph nodes at final pathology

Postoperative histology is reported in Table 3. The total number of harvested lymph nodes per patient was 32 (IQR 26–41), with a median of 1 (IQR 0–1) lymph node harvested after NIR. Figure 2 shows the harvested stations after NIR visualization, as well as whether lymph nodes were found at final pathology. In Table 4, the possibility of finding nodal tissue with NIR visualization and the possibility of retrieving nonnodal tissue per patient is tested against different baseline and pathological characteristics.

**Table 3:** Postoperative histology

	*N* = 18
Pathological stage	
0	3 (16.7)
IA	2 (11.1)
IB	2 (11.1)
IIA	1 (5.6)
IIB	5 (27.8)
IIIA	2 (11.1)
IIIB	2 (11.1)
IIIC	1 (5.6)
pT	
T0	3 (16.7)
T1	2 (11.1)
T2	3 (16.7)
T3	9 (50.0)
T4	1 (5.6)
pN	
N0	8 (44.4)
N1	5 (27.8)
N2	1 (5.6)
N3a	3 (16.7)
N3b	1 (5.6)
*Lymph nodes*	
Total	32 (26–41)
Before ICG	31 (26–40)
After ICG	1 (0–1)
Metastatic	1 (0–5)
Before ICG	1 (0–5)
After ICG	0 (0–0)
Response > 1	7 (38.9)
Patients with metastatic nodes at ICG	2 (11.1)
Grading	
Well differentiated	2 (11.1)
Moderately differentiated	9 (50.0)
Poorly differentiated	3 (16.7)
Missing	4 (22.2)
Postoperative location	
Body	10 (55.6)
Antrum	8 (44.4)
Postoperative histology	
Intestinal adenocarcinoma	5 (27.8)
Diffuse adenocarcinoma	7 (38.9)
Mixed adenocarcinoma	1 (5.6)
Adenocarcinoma with neuroendocrine features	1 (5.6)
No residual tumor	4 (22.2)

Data are number (percentage) and median (interquartile range)

**Table 4:** Correlation of baseline and pathological characteristics with the finding of nodal tissue with NIR, or with the retrieval of non-nodal tissue

	Retrieval of additional nodes (10 pts)	P*	Retrieval of non-nodal tissue (6 pts)	P*
Age at surgery (year)		0.596		0.180
≤ 65 (8 pts)	5 (62.5)		4 (50.0)	
> 65 (10 pts)	5 (50.0)		2 (20.0)	
Gender		0.180		0.494
Female (11 pts)	7 (63.6)		3 (42.9)	
Male (7 pts)	3 (42.9)		3 (27.3)	
BMI		0.04		0.317
< 25 (9 pts)	8 (88.9)		2 (22.2)	
≥ 25 (9 pts)	2 (22.2)		4 (44.4)	
Tumor location		1		1
Body (9 pts)	5 (55.6)		3 (33.3)	
Antrum (9 pts)	5 (55.6)		3 (33.3)	
Neoadjuvant CT		0.748		0.468
No (8 pts)	5 (71.4)		3 (42.9)	
Yes (11 pts)	5 (45.5)		3 (27.3)	
Pathologic T stage		0.671		0.737
T1–T2 (8 pts)	4 (50.0)		3 (37.5)	
T3–T4 (10 pts)	6 (60.0)		3 (30.0)	
Cases		0.343		0.317
First half (9 pts)	6 (66.7)		2 (22.2)	
Second half (9 pts)	4 (44.4)		4 (44.4)	

*Chi-square test

*pts* patients

### Postoperative Outcomes

The median length of hospital stay (Supplementary Table 1) was 10 days (IQR 9–14). Ninety-day complications occurred in three (17%) patients: one bile leak in the cholecystectomy bed that required reoperation, one chylous fistula that was managed conservatively, and one duodenal stump leak that resulted in death. The remaining 17 patients are alive after a median follow-up of 21 months. Ten (56%) patients received adjuvant chemotherapy. Recurrence occurred in four (22%) cases: two locoregional, one peritoneal, and one distant. All patients who recurred were nodal positive at final pathology, except one who had no evidence of residual disease. The median time to recurrence was 10.5 (IQR 5–15) months.

## Discussion

The Greeneye study was designed to assess whether NIR visualization after peritumoral ICG injection improves the effectiveness of laparoscopic lympadenectomy by increasing the number of retrieved lymph nodes. Preoperative endoscopy performed with the only goal of ICG peritumoral injection poses several organization, economic and patients’ related concerns. For this reason, we designed the study with a Simon optimal two-stage design, so that the trial could be stopped after the first eight cases in case of futility. Because retrieval of nonnodal tissue (nodal noncompliance) has been repeatedly reported in NIR-guided lymphadenectomy, we considered nodal noncompliance as a potentially harmful event, and in each patient NIR was considered useful only if the number of additional retrieved nodes was higher than the number of nonnodal tissues (response > 0). Overall, response was > 0 in 7 cases (38.9%): the null hypothesis of having < 20% patients with response > 0 was rejected, and the study confirmed that NIR visualization after ICG injection might positively influence the quality of lymphadenectomy in laparoscopic subtotal gastrectomy for cancer.

Several studies, and a recent meta-analysis, suggest that fluorescence guided LND allows for a higher number of LNs harvested with respect to standard LND, possibly reducing intraoperative blood loss and without impact on operative time and postoperative complications.^16^ Most of these studies (mainly comparative retrospective, only two randomized trials) compare ICG-guided LND to standard LND; allocation to either group is clearly not blinded, and the reasons for a better performance of ICG-guided LND in terms of number of retrieved LNs might be related to different factors that are not necessarily related to the effectiveness of fluorescence guidance. In this study, to overcome this potential bias, LND was performed as per local standards and NIR visualization was used after specimen extraction, in order to appreciate its crude effectiveness. We chose a Simon’s two-stage, phase II design as we sought to use a composite endpoint of efficacy that would consider nonnodal tissue retrieval as a negative event. Few studies have been published in the setting of ICG-guided LND with this approach, and no data on the safety/efficacy per patient were available: such a design allowed us to interrupt the study early in case of futility. We decided to restrict our population only to patients undergoing laparoscopic distal gastrectomy in order to have a homogeneous study population. The median number of additional nodes that were retrieved after NIR visualization was approximately 1, resulting in an increase of only 2.3% in the total number of nodes retrieved. This result is in contrast with the previously cited meta-analysis (mean 7.4 additional LNs with ICG guidance)^16^ and with the two available, randomized, controlled trials that respectively reported a mean number of 8.5 and 7.9 additional LNs.^12,17^ As said before, this difference can be partially explained by the fact that in our study fluorescence was used only at the end of lymphadenectomy, when the procedure had already been judged as adequate by the operating surgeon. This result is not influenced by a performance bias. Moreover, patients in this series had a higher body mass index (BMI) than in most of the previous studies, and more than 60% received neoadjuvant chemotherapy, both factors with a known negative impact on the number of retrieved LNs.^18,19^

From a clinical standpoint, in this series NIR visualization and the resulting nodal retrieval did not have a significant impact overall: neither appropriateness of lymphadenectomy (in terms of LNs retrieved) nor staging (in terms of metastatic nodes) was affected by the additional removal of fluorescent stations. However, in one patient the fluorescent station that was harvested resulted in the fourth metastatic node (more than 47 LNs retrieved), potentially changing the curative impact of the surgical procedure; in four patients fluorescent staining with nodal tissue was found outside the D2 area (4sa, 11d, and 13), potentially representing an unexpected area of tumor drainage. Because no stage migration nor increased adequacy of lymphadenectomy were achieved, whether the statistical benefit of the procedure resulted in a clinically meaningful benefit remains uncertain.

We further analyzed our data to check whether any patient or tumor related characteristic was associated with the possibility of retrieving additional nodal tissue thanks to ICG staining. Interestingly, as shown in Table 3, we found that additional nodal tissue was retrieved only in two of the nine patients (22.2%) with a BMI > 25, whereas in nearly 90% of patients with BMI < 25. This could be related both to a different spread of ICG in patients with more represented adipose tissue and to a lower visualization of NIR fluorescence in patients with higher BMI. However, this result is in line with the finding that NIR visualization results in a significantly higher number of nodes retrieved in the Eastern population, while it is less reproducible in the Western one.^20^

We found that, after standard lymphadenectomy was performed, the LN station that was most frequently fluorescent, thus most commonly harvested with NIR visualization, was station 6. Lymphadenectomy in the infrapyloric area is considered a challenging procedure because of the conformation of this area and needs high anatomical knowledge and surgical expertise to be performed systematically. When LN dissection is not performed correctly along the avascular planes, LN retrieval may be inadequate. Previous studies showed that ICG fluorescence during laparoscopic gastrectomy facilitates lymphadenectomy in the subpyloric region and might allow for a higher number of harvested LNs in this area.^21,22^ Our results confirm these findings: because the infrapyloric region is rich in lymphatic tissue and a major drainage site in distal gastric cancer, the use of ICG allowed us to obtain a complete lymphadenectomy with a subsequent potential prognostic advantage. It has to be noted, however, that further lymph nodal harvesting at the infrapyloric region occurred only in the first ten cases, whereas in the remaining patients, no fluorescent tissue at station 6 was observed at the end of lymphadenectomy. It is possible that, while all procedures were performed by experience surgeons, the NIR visualization of station 6 in the first cases NIR visualization allowed for a “learning curve” on a more radical lymphadenectomy of the infrapyloric region, leading to a wider upfront dissection of this region. In this sense, the usefulness of NIR visualization would disappear once this knowledge had been acquired; however, this suggests that ICG may be used as a quality control for lymphadenectomy appropriateness if the procedure is performed by trainees or surgeons at the beginning of their learning curve in laparoscopic gastrectomy.

This study has some limitations. First, the number of patients is small; even if the primary endpoint was met, the generalizability of results might be limited by the relative heterogeneity of tumor stages. Second, in this study, we did not observe any complication directly correlated with the EGDS and the preoperative submucosal injection of ICG, nor resulting from the retrieval of fluorescent tissue at the end of LND. Analyses on long-term oncological outcomes could not be performed due to small sample size and insufficient follow-up. The time spent for the NIR-guided retrieval of additional nodal stations was 12 (range 8–16) minutes, which appeared to minimally impact on the total operative time as observed in most studies.^16^ However, studies targeted to evaluate the intention to treat risks of this procedure, and the economic justification, are still needed. Finally, this study was targeted to assess the clinical benefit of NIR in distal gastrectomy, and its magnitude in total gastrectomy might be different. As observed elsewhere, D2 lymphadenectomy of distal gastrectomy is less difficult, and intraoperative lymph node compliance is easier to achieve with respect to total gastrectomy.^17^

## Conclusions

This study confirms that preoperative ICG injection and subsequent NIR visualization might improve the quality of lymphadenectomy during laparoscopic surgery for gastric cancer without causing harm. However, in presence of adequate and well-performed D2 lymphadenectomy, the true benefit of additional NIR-guided lymphadenectomy seems limited, barely justifying the diffusion of the technique. Further evidence is needed to assess a true oncological benefit of ICG-guided lymphadenectomy, also considering the logistical and financial downsides of preoperative ICG injection.

## Supplementary Information

Below is the link to the electronic supplementary material.

Supplementary file1 (DOCX 2792 KB)
