# Supplementary material for: Impact of Indocyanine Green Fluorescence Imaging on Lymphadenectomy Quality During Laparoscopic Distal Gastrectomy for Gastric Cancer (Greeneye): An Adaptative, Phase 2, Clinical Trial
Source: Ann Surg Oncol. 2023 Jul 13;30(11):6803–11. doi: 10.1245/s10434-023-13848-y (PMC10506942; doi:10.1245/s10434-023-13848-y)

**SUPPLEMENTARY MATERIAL**

| **Table 1. Operative and postoperative data (stage I-II)** | |
| --- | --- |
|  | **N = 18** |
| **Laparoscopic distal gastrectomy** | 18 (100) |
| **D2 lymphadenectomy** | 18 (100) |
| **Associated cholecystectomy** | 5 (28) |
| **Time between ICG injection and intraoperative NIR visualization (hours)** | 22.5 (22 – 23) |
| **Operative time (mins)** | 316 (294 – 329) |
| **Time for additional nodal retrieval (mins)** | 12 (8-16) |
| **Any ICG visualization**  *D2 territory*  *Outside D2* | 14 (78)  13 (72)  6 (33) |
| **Length of stay (days)** | 10 (9 – 14) |
| **90-day postoperative complications**  *Anastomotic leak*  *Delayed gastric emptying*  *Chyle leak*  *Biliary fistula*  *Anemia requiring transfusion*  *Pulmonary complication* | 6 (33)  2 (11)  2 (11)  1 (6)  1 (6)  1 (6)  1 (6) |
| **Max Clavien dindo grade**  I  II  IIIb  V | 1 (6)  3 (17)  1 (6)  1 (5.5) |
| **Adjuvant chemotherapy** | 10 (56) |
| **Alive at latest follow up**  *Cancer-related deaths* | 15 (83)  2 (11) |
| **Recurrence**  *Locoregional*  *Peritoneal*  *Distant metastases* | 4 (22)  2 (11)  1 (6)  1 (6) |
| Data are number (percentage) and median (interquartile range) | |

Figure 1. Specimen examination after extraction


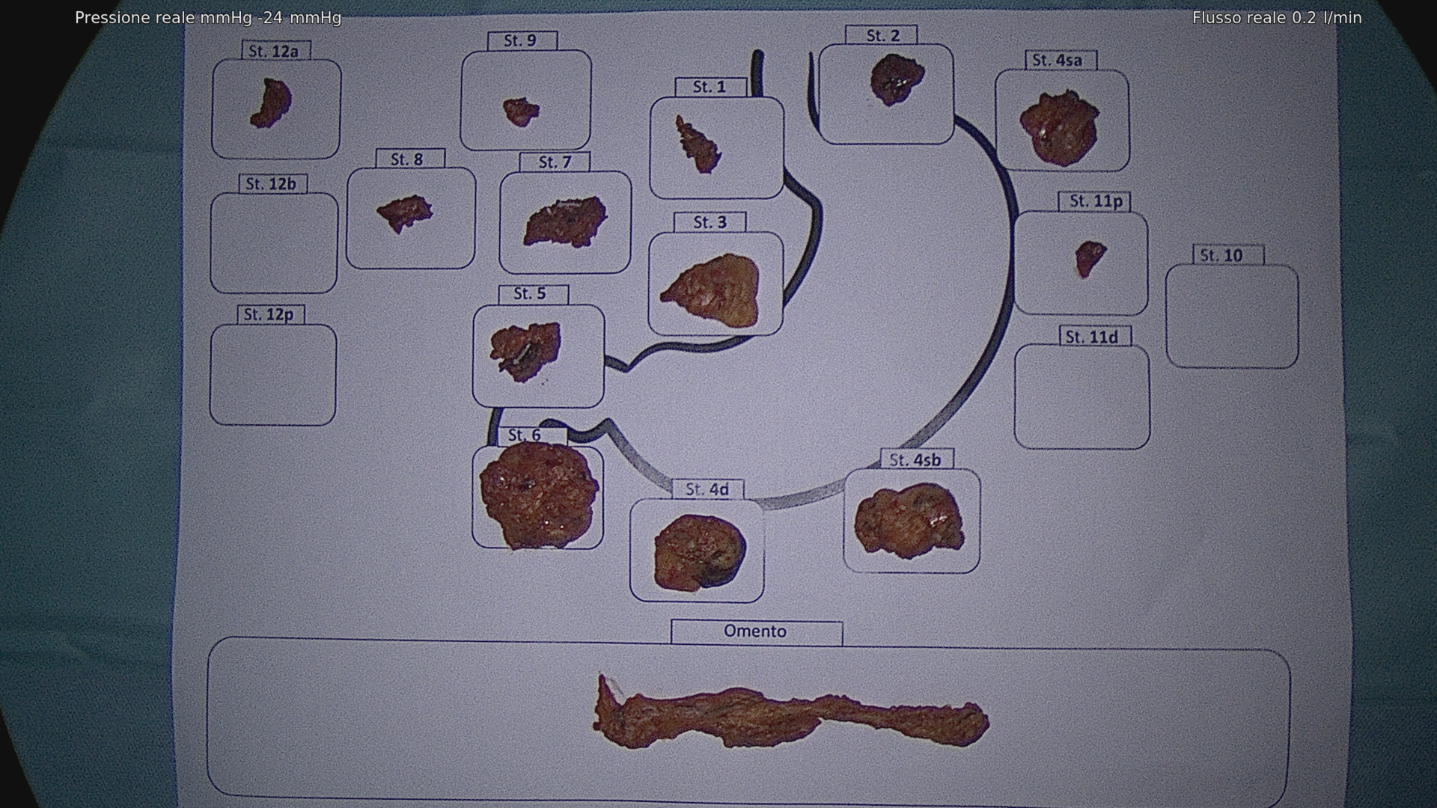

Supplement: Supplementary file 1 — Supplementary file1 (DOCX 2792 KB) [file 10434_2023_13848_MOESM1_ESM.docx]
